# Implications of PI3K/AKT inhibition on REST protein stability and neuroendocrine phenotype acquisition in prostate cancer cells

**DOI:** 10.18632/oncotarget.19386

**Published:** 2017-07-19

**Authors:** Ruiqui Chen, Yinan Li, Ralph Buttyan, Xuesen Dong

**Affiliations:** ^1^ Vancouver Prostate Center, Department of Urologic Sciences, The University of British Columbia, Vancouver, Canada

**Keywords:** PI3K/AKT inhibition, REST degradation, neuroendocrine differentiation, neuroendocrine prostate cancer

## Abstract

Treatment-induced neuroendocrine prostate cancer (t-NEPC) is an aggressive subtype of prostate cancer (PCa) that arises as a consequence of rigorous androgen receptor (AR) pathway inhibition (ARPI) therapies. While the PI3K/AKT pathway has been investigated as a co-therapeutic target with ARPI for advanced PCa, whether this strategy can prevent tumor progression to t-NEPC remains unknown. Here, we report that PI3K/AKT inhibition alone reduces RE-1 silencing transcription factor (REST) protein expression and induces multiple NE markers in PCa cells. The loss of REST by PI3K/AKT inhibition is through protein degradation mediated by the E3-ubiquitin ligase β-TRCP and REST phosphorylations at the S1024, S1027, and S1030 sites. Since AR inhibition can also deplete REST, the combinational inhibition of PI3K/AKT and AR further aggravated REST protein reduction. We profiled the transcriptomes of AKT and AR inhibitions in the LNCaP cells. The Gene Set Enrichment Analysis (GSEA) showed that these transcriptomes are highly correlated with the REST-regulated gene signature. Co-targeting AKT and AR resulted in a higher correlation comparing to those of single treatment. Comparing these transcriptomes to the t-NEPC gene signature in patients by GSEA, we observed that adding AKT inhibition to AR blockade enhanced the expression of neurogenesis-related genes and resulted in a stronger and broader upregulation of REST-regulated genes specific to t-NEPC. These results indicate that AKT pathway inhibition can induce neuroendocrine differentiation of PCa cells *via* REST protein degradation. It delineates a potential risk for the AR and PI3K/AKT co-targeting strategy as it may further facilitate t-NEPC development.

## INTRODUCTION

Androgen receptor pathway inhibition (ARPI) can prolong the survival for patients with advanced, locally recurrent, or metastatic prostate cancer (PCa); however, relapse to ARPI-resistant disease, referred to as castration resistant prostate cancer (CRPC), is inevitable [1]. This resistance is often associated with reactivation of survival signaling cascades to cope with cellular stress caused by ARPI [1, 2]. The PI3K/AKT signaling pathway activation is one of the key survival pathways associated with ARPI resistance [3]. This signaling pathway is targeted for its importance in promoting tumor progression and resistance for therapy-induced cell death [4]. In fact, genes within this pathway bearing genomic and transcriptional alterations that result in overactive AKT signaling have been identified in almost all PCa at advanced stages [5, 6]. More importantly, the PI3K/AKT pathway has been reported to have a reciprocal feedback activation mechanism with AR, resulting in further overactive AKT signaling upon AR inhibition in PTEN-deficient PCa cells [7-9]. These findings together build a strong rationale for co-targeting the PI3K/AKT and AR pathways in order to achieve a better outcome for PCa patients. Multiple clinical trials utilizing this co-targeting strategy have been conducted to investigate the efficacy of this novel combination treatment (ClinicalTrials.gov).

However, whether co-targeting the PI3K/AKT and AR signaling can have unexpected effects as a facilitator of treatment-induced neuroendocrine prostate cancer (t-NEPC) remains unknown. t-NEPC is one of the most lethal subtypes of castration-resistant prostate cancer (CRPC). The median survival time of t-NEPC is approximately 7 months upon diagnosis due to the lack of early detection method and treatment options besides systemic chemotherapy [10]. Although NEPC is rare as the primary form of PCa with a prevalence of less than 1%, t-NEPC is estimated to consist up to 25% in CRPC patients status post first- and second-line of anti-AR therapies [11]. Emerging studies indicate that the rise of t-NEPC prevalence is most likely a consequence of neuroendocrine transdifferentiation of adenocarcinoma under the selection pressure of anti-AR therapies [12]. Therefore, the incidence of t-NEPC is expected to be more prevalent with the widespread application of more potent AR inhibitors to PCa patients.

To date, the molecular mechanisms by which adenocarcinoma progresses into t-NEPC under anti-AR therapies remain to be fully elucidated. Currently, a variety of genetic factors, including P53 and Rb1 loss, N-Myc amplification, mitotic deregulation *via* AURKA, alternative splicing by serine/arginine repetitive matrix4 (SRRM4), BRN2 upregulation, and REST loss appear to have a role [13-20]. In particular, loss of the RE-1 silencing transcription factor (REST), a master negative regulator of neurogenesis [21], is one of the hallmarks of t-NEPC development [22, 23]. The REST protein is a 1097-amino acid transcription repressor that binds to the 21-bp repressor element 1 (RE-1) normally located within the regulatory region of target genes [24]. REST is highly expressed in embryonic stem cells and non-neuronal cells, where it acts as a negative master regulator of neurogenesis. Loss of REST allows de-repression of genes required for neural cell differentiation [25]. The expression of REST is regulated at both the RNA and protein levels. At the RNA level, the REST gene can undergo alternative splicing mediated by RNA-splicing factors such as the SRRM4 to generate a dominant negative form of REST4 [19, 26]. At the protein level, REST protein is tightly regulated at the post-translational level by ubiquitination and deubiquitinating processes [27]. β-TRCP is an F-box E3 ligase that recognizes phosphorylated REST protein for ubiquitination and proteasome degradation [28, 29]. Serine residuals at 1024, 1027 and 1030 are key to determine REST protein stability [28]. In contrast, HAUSP (the herpesvirus-associated ubiquitin-specific protease, also known as USP7) had been shown to suppress REST degradation through a deubiquitination process [30].

In this study, we report an unexpected effect of PI3K/AKT inhibition in the context of t-NEPC. Here we show that PI3K/AKT inhibition can reduce REST protein expression through ubiquitination and subsequently increase NE markers in multiple PCa cell lines. Combined AKT and AR inhibition aggravated REST depletion and accelerated NE transdifferentiation in PCa cells. Our findings indicate the potential for an unexpected complication of a combined PI3K/AKT and AR targeting strategy for PCa patients.

## RESULTS

### PI3K/AKT inhibition downregulates REST expression and induces NE markers in PCa cells

**Figure 1:**
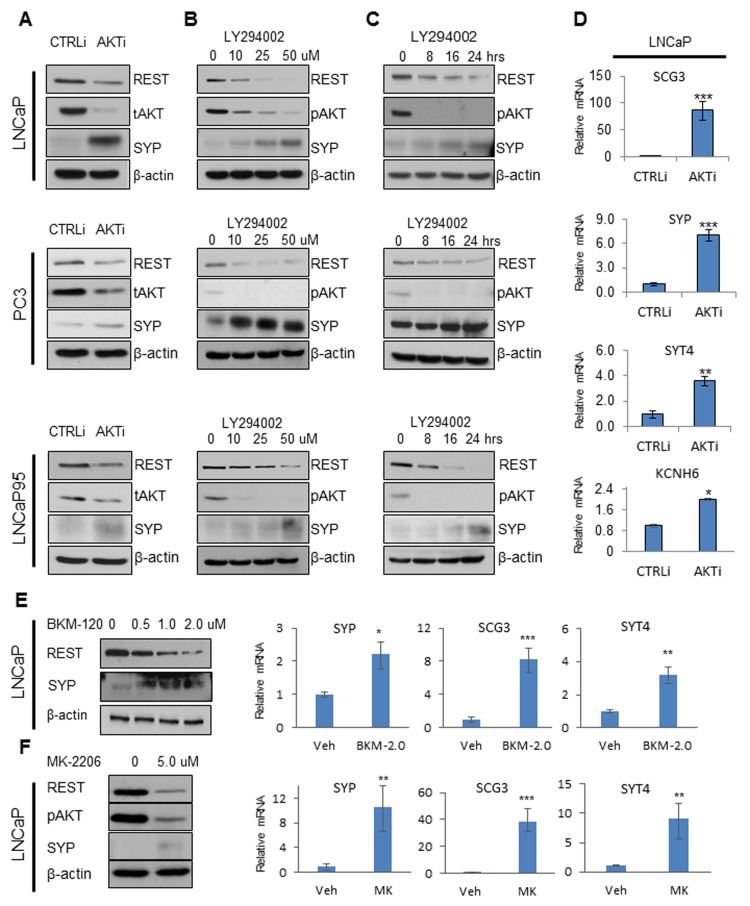
PI3K/AKT inhibition reduces REST and increases NE markers **A.** LNCaP, PC3, and LNCaP95 cells were transfected with control siRNA (CTRLi) or siRNA against AKT (AKTi) for 48 hours. Protein levels of REST, total AKT (tAKT), SYP, and β-actin were measured by immunoblotting. **B.** LNCaP and LNCaP95 cells were treated with 0, 10, 25, 50 μM LY294002 for 24 hours. PC3 cells were treated with 0, 50, 100, 150 μM LY294002 for 24 hours. **C.** LNCaP and LNCaP95 cells were treated with 50 μM LY294002 and PC3 cells were treated with 100 μM LY294002 for 0, 8, 16, 24 hours. Cell lysates were immunoblotted with antibodies against REST, pAKT, SYP, and β-actin. **D.** Relative mRNA levels of neuroendocrine markers from LNCaP cells treated with CTRLi or AKTi for 48 hours were measured by real-time PCR. Statistical analyses were performed by paired student’s *t*-test with *p* < 0.05 as *, *p* < 0.01 as ** and *p* < 0.001 as ***. LNCaP cells were treated with 0, 0.5, 1.0, 2.0 μM BKM-120 **E.** and with 0, 5 μM MK-2206 **F.** for 24 hours. Immunoblotting and real-time PCR were performed to test the expression of genes of interest.

Human prostate cancer cell lines such as LNCaP, PC3, and LNCaP95 cells are PTEN-deficient and have overactive PI3K/AKT signaling as is frequently observed in metastatic PCa. To test whether AKT inhibition can affect REST expression and induce an NE-phenotype in PCa cells, we transiently transfected each of them with control and AKT siRNA. AKT depletion in all cell lines resulted in downregulation of REST protein and upregulation of the NE marker synaptophysin (SYP) (Figure 1A). Western blots further confirmed that REST depletion in LNCaP, PC3, and LNCaP95 cells by siRNA induced both SYP and neuron-specific enolase (NSE) levels (Supplementary Figure S1). Likewise, when LNCaP, PC3, and LNCaP95 cells were treated with the PI3K inhibitor, LY294002, the expression of REST was suppressed while SYP protein levels increased in both time- and does-dependent manners (Figure 1B & 1C). The treatment of LY294002 in LNCaP cells in fact increased the expression of SYP in most cells according to immunofluorescence results (Supplementary Figure S2). Indeed, AKT depletion by siRNA induced a range of NE markers such as SCG3, SYT4, and KCNH6 (*p* < 0.05) in LNCaP cells at the mRNA level (Figure 1D). Similarly, the PI3K inhibitor BKM-120 and AKT inhibitor MK-2206 also reduced REST and increased NE expressions in LNCaP cells (Figure 1E&1F). Collectively, our results show that PI3K/AKT inhibition can downregulate REST protein expression and induce NE markers in PTEN-deficient PCa cells.

### PI3K/AKT inhibition reduced REST protein stability

**Figure 2:**
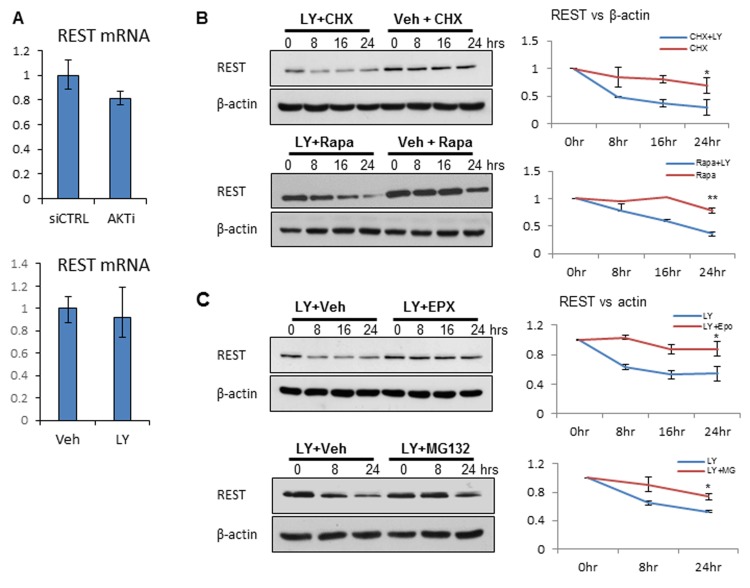
PI3K/AKT inhibition affects REST protein stability **A.** LNCaP cells were transfected with CTRLi or AKTi for 48 hours (top) and vehicle (Veh) or 50 μM LY294002 (LY) for 24 hours (bottom). Relative REST mRNA levels were measured by real-time PCR. **B.** LNCaP cells were treated with 100 μg/ml cyclohexamide (CHX) or 200 nM rapamycin (Rapa) in the condition of vehicle or LY294002 for 0, 8, 16, 24 hours. **C.** LNCaP cells were treated with 50 μM LY294002 in the condition of vehicle or 100 nM epoxomicin (EPX) for 0, 8, 16, 24 hours. LNCaP cells were also treated with 50 μM LY294002 plus vehicle or 8 μM MG132 for 0, 8, 16 hours. Cell lysates were immunoblotted with antibodies against REST and β-actin. Experiments were repeated at least three times and one set of the representative blots was shown. Densitometry analyses of REST to β-actin ratios were performed by the Image J software and plotted as mean+SEM. Statistical analyses were performed by paired student’s *t*-test with *p* < 0.05 as * and *p* < 0.01 as **.

Although REST protein levels were reduced by AKT knockdown or inhibition, mRNA levels for REST were unchanged, suggesting that suppression of REST expression by PI3K/AKT inhibition is at the post-transcriptional level (Figure 2A). While treatment of LNCaP cells with translation inhibitors including cycloheximide (CHX) or rapamycin (Rapa) [31] for up to 24h did not significantly reduce REST protein levels, the addition of LY294002 did accelerate REST protein reduction, indicating that the effect of PI3K/AKT blockade is likely at the post-translational level (Figure 2B). To test whether REST protein reduction by PI3K/AKT inhibition is mediated through the proteasome pathway, LNCaP cells were treated with proteasome inhibitors including epoxomicin (EPX) and MG132 (Figure 2C). Additions of these proteasome inhibitors reversed LY294002-induced REST reduction. These findings were further validated in PC3 and LNCaP95 cells (Supplementary Figure S3A). Together, these results suggest that PI3K/AKT inhibition affects REST protein stability *via* a proteasome-mediated pathway.

### REST protein degradation by PI3K/AKT inhibition is mediated through ubiquitination

**Figure 3:**
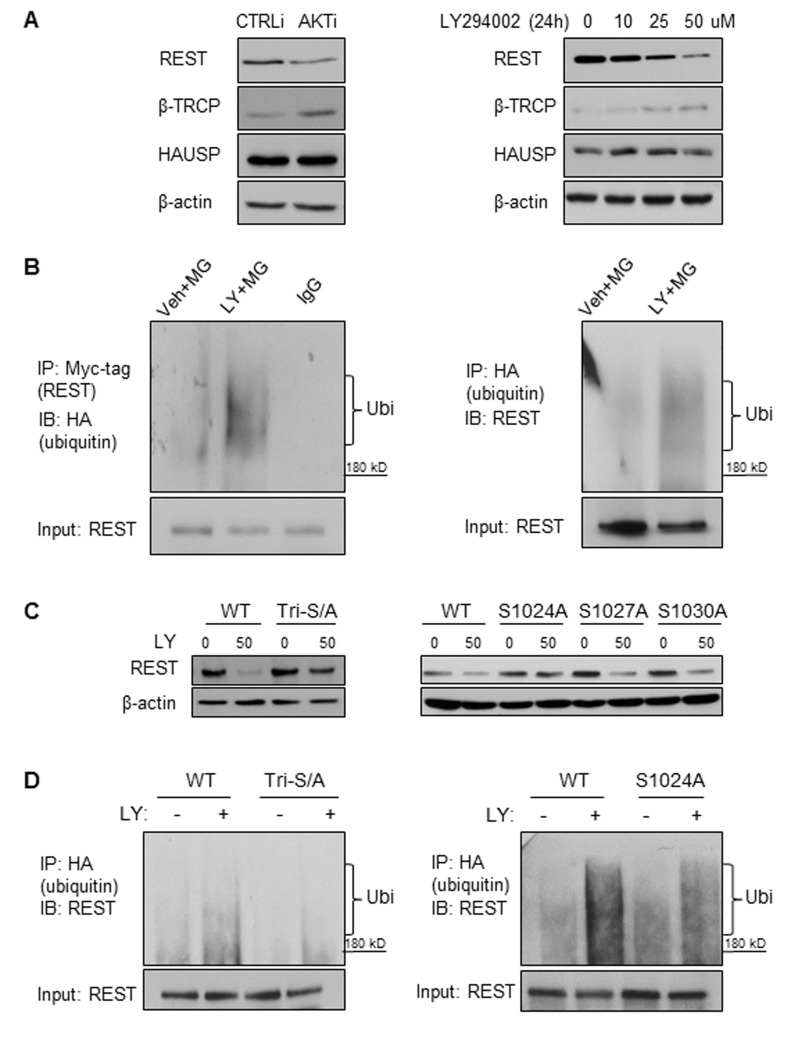
REST protein was ubiquitinated under PI3K/AKT inhibition mediated by β-TRCP and REST phospho-degron **A.** LNCaP cells were transfected with CTRLi or AKTi for 48 hours (left) or treated with 0, 10, 25, 50 μM LY294002 for 24 hours (right). Cell lysates were collected and REST, β-TRCP, HAUSP and β-actin protein levels were measured by immunoblotting. **B.** On the left: LNCaP cells were transfected with myc-tagged REST and HA-tagged ubiquitin for 48 hours followed by 8 μM MG132 plus vehicle or 50 μM LY294002 treatment for 8 hours. Cell lysates were immunoprecipitated with the myc-tag antibody followed by immunoblotting of HA-tags for ubiquitinated-REST detection. On the right: LNCaP cells were transfected with HA-tagged ubiquitin for 48 hours followed by the 8 μM MG132 plus vehicle or LY294002 treatment for 8 hours. Cell lysates were immunoprecipitated with the HA-tag antibody and immunoblotted with the anti-REST antibody. **C.** LNCaP cells were transfected with the flag-tagged wild type REST (WT) or the flag-tagged REST with the S1024/1027/1020A triple mutation (Tri-S/A) for 24 hours, then treated in the condition of vehicle or 50 μM LY294002 for 24 hours (left). LNCaP cells were also transfected with REST (WT) or REST mutant (either S1024A, S1027A, or S1030A) for 24 hours followed by the treatment of vehicle or 50 μM LY294002 for 24 hours (right). Cell lysates were immunoblotted with antibodies against REST and β-actin. **D.** LNCaP cells transfected with REST (WT) or REST (Tri-S/A) (left) and REST (WT) or REST (S1024A) (right) for 48 hours followed by treatment of 8 μM MG132 plus vehicle or 50 μM LY294002 for 8 hours. Cell lysates were immunoprecipitated with the HA-tag antibody and immunoblotted with the REST antibody.

β-TRCP and HAUSP are the most well-described E3-ubiquitin ligase and deubiquitinase that determines REST protein stability, respectively [27]. Western blot results showed that AKT siRNA and LY294002 both increased β-TRCP expression, but did not affect HAUSP protein levels (Figure 3A). These results suggest that increased β-TRCP E3-ligase expression upon PI3K/AKT inhibition may induce REST ubiquitination and subsequent protein degradation. To test this hypothesis, we transfected cells with vectors encoding myc-tagged REST and HA-tagged ubiquitin and treated the cells with MG132 and LY294002. *In vitro* ubiquitination assays showed that REST ubiquitination was increased by LY294002 in LNCaP cells (Figure 3B) as well as in PC3 and LNCaP95 cells (Supplementary Figure S3B). To test whether phosphorylation of REST regulates its protein degradation by PI3K/AKT inhibition, we transfected LNCaP cells with vectors encoding wild type REST or a mutated REST cDNA (tri-serine mutation and single mutations at serine 1024, 2017 and 1030) (Figure 3C). These mutations were known to suppress the ability of β-TRCP to ubiquitinate REST protein [28]. Immunoblotting showed that REST protein degradation was largely rescued by the triple mutation and was partially rescued by the S1024A mutation. However, it is interesting to note that REST function may be compromised in the “non-degradable” form as shown by Western blot and luciferase assays (Supplementary Figure S4). Regardless*, in vitro* ubiquitination assays further confirmed that LY294002-mediated REST ubiquitination was reduced by the REST triple mutation, and to a lesser extent by the REST S1024A single mutation (Figure 3D). These findings support the idea that reduction of REST by PI3K/AKT inhibition require phosphorylations within the 1024-1030 region, followed by β-TRCP mediated REST ubiquitination and protein degradation.

### Combinational effects of PI3K/AKT and AR inhibitions in PCa cells and t-NEPC patients

**Figure 4:**
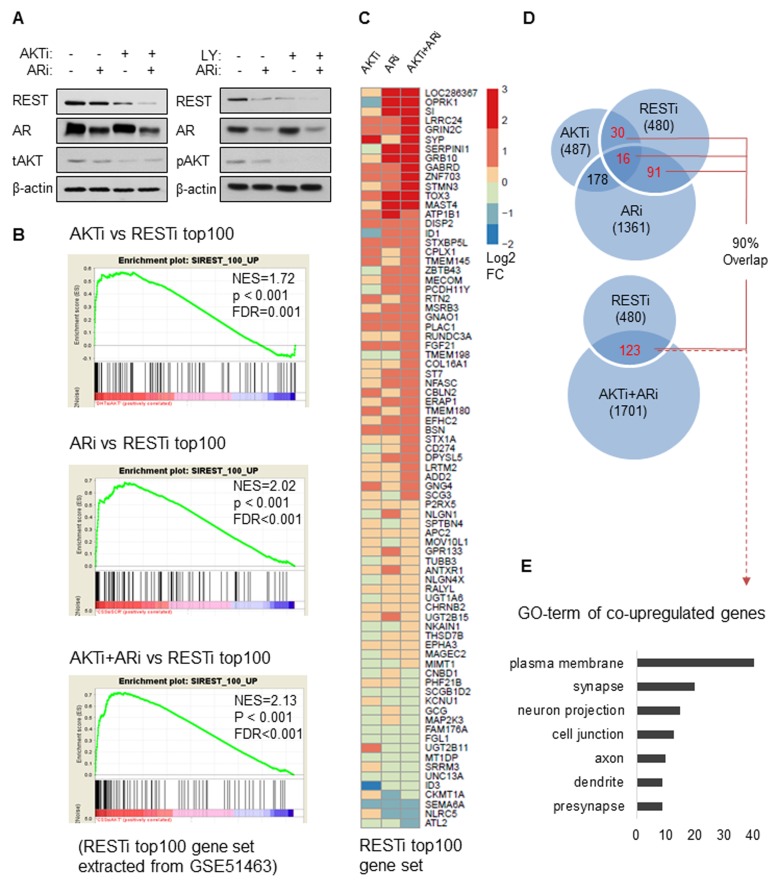
REST degradation with AKTi and ARi combination treatment **A.** On the left: LNCaP cells were cultured in RPMI containing FBS for 48 hours followed by transfections of control siRNA or siRNA against AKT (AKTi) for 48 hours. LNCaP cells were also cultured in the androgen depletion condition in phenol red-free RPMI containing 5% CSS for 48 hours and then transfected with control siRNA (ARi) or siRNA against AKT for 48 hours (AKTi+ARi). On the right: LNCaP cells were cultured in RPMI containing FBS or CSS for 72 hours followed by 50 μM LY294002 or vehicle treatment for 24 hours. Cell lysates were immunoblotted for antibodies against REST, AR, tAKT, pAKT, and β-actin. **B.** Transcriptomes of LNCaP cells treated with control, AKTi, ARi, and AKTi+ARi were analysed by Ampliseq Transcriptome Analysis. Differential gene expressions of AKTi, ARi, and AKTi+ARi comparing to the control were analyzed by the DESeq2 package in R. GSEA enrichment plots showed the correlations of AKTi, ARi, or AKTi+ARi with the top 100 upregulated genes from the LNCaP cells with REST silencing (RESTi) obtained from the GEO database (GSE51463). Top 100 upregulated genes were ranked according to the log2 fold change and filtered by padj < 0.1 after Benjamini-Hochberg multiple testing correction. **C.** A heatmap representing expression changes of genes in the RESTi top100 gene set after the treatment of AKTi, ARi, or AKTi+ARi. **D.** A Venn diagram depicting the co-upregulated genes (fold change > 1.5 and padj < 0.1) between each of the AKTi, ARi, or AKTi+ARi treatment and RESTi. 90% of co-upregulated genes between RESTi and each of the single treatment were also found within the co-upregulated genes between RESTi and AKTi+ARi. **E.** The co-upregulated genes between AKTi+ARi and RESTi (*n* = 123) were analysed by DAVID (version 6.7). Top ranked GO_TERM sorted gene groups were listed.

Previous studies have shown that REST protein expression can be reduced by AR inhibition [19, 20, 22] and we confirmed this in LNCaP cells (Supplementary Figure S5). As we have shown that PI3K/AKT inhibition can reduce REST *via* protein degradation, we tested the effects of combination AR/AKT inhibitory treatment. LNCaP cells were transfected with AKT siRNA (AKTi), cultured in androgen depletion condition as in phenol red-free RPMI medium containing 5% CSS (ARi), or AKTi+ARi. Western blot results showed that while each treatment separately suppressed REST protein expression, combination treatment resulted in a more effective depletion of REST protein (Figure 4A). Combined ARi treatment with LY294002 had the same effect. We then profiled the transcriptomes of LNCaP cells treated with vehicle, AKTi, ARi or AKTi+ARi using the Ampliseq Transcriptome Analysis [32] as described in Materials and methods. Comparison of the gene profiles of the treatment groups to the transcriptome of LNCaP cells treated with REST siRNA (GEO database GSE51463) [20] using Gene Set Enrichment Analysis (GSEA) [33] revealed that both the AKTi and ARi treated transcriptomes were all highly correlated with the top ranked 100 genes regulated by REST (FDR = 0.001 and < 0.001 respectively) (Figure 4B). Reverse GSEA analyses further confirmed that the transcriptome of RESTi was also correlated with the top 100 upregulated genes in each of the AKTi, ARi, and AKTi+ARi conditions (Supplementary Figure S6). Heatmapping showed that the combination treatment (AKTi+ARi) not only increased the diversity, but also the fold changes of REST-regulated genes (Figure 4C). For example, some genes, exemplified by OPRK1 were upregulated by ARi but not by AKTi whereas some genes, exemplified by SYP were upregulated by AKTi but not ARi. These types of discordant genes were, however, all upregulated by the combination of AKTi+ARi. Finally, genes such as LRRC24, GRIN2C, GABRD showed stronger fold changes upon AKTi+ARi treatment compared to single treatment conditions. Similarly, among the significantly upregulated genes (fold change > 1.5 and padj < 0.1), the AKTi+ARi group had more co-upregulated genes with the RESTi group (*n* = 123) than the AKTi (*n* = 46) or ARi (*n* = 107) groups (Figure 4D). Interestingly, 90% of the co-upregulated genes shared by RESTi with each of the single treatments were also co-upregulated by RESTi and AKTi+ARi. Gene Ontology (GO) analyses showed that the 123 genes co-targeted by AKT, AR and REST are associated with cellular functions such as plasma membrane, synapse, neuron projection and cell junction (Figure 4E), suggesting that these genes may indicate the t-NEPC transdifferentiation in cells treated by AKT and AR inhibitions. In summary, these results suggest that AKTi alone can induce NE phenotypes through REST down-regulation, and the combination of AKTi and ARi can more stringently reduce REST protein levels with concomitant increased expression of REST-regulated genes in LNCaP cells.

**Figure 5:**
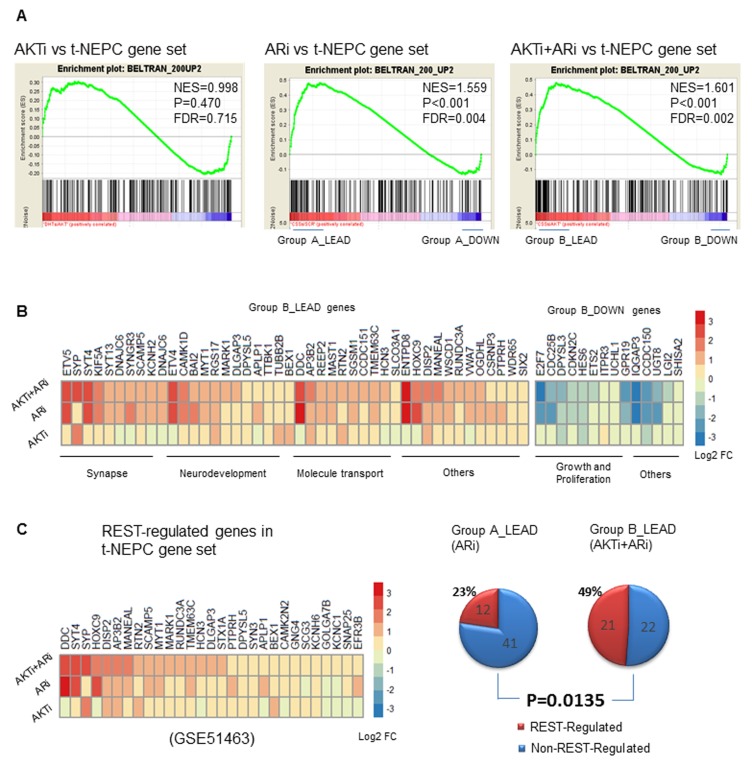
AKTi, ARi, and AKTi+ARi treatment in relation to t-NEPC patients **A.** GSEA enrichment plots showing the correlations of AKTi, ARi, or AKTi+ARi with the t-NEPC gene set that consisted of the top 200 upregulated genes from t-NEPC patients from the Beltran cohort. [12] Top 200 upregulated genes were ranked according to the log2 fold change and filtered by padj < 0.05 after Benjamini-Hochberg multiple testing correction. **B.** The strongly positively correlated leading edge group genes (*n* = 43) as well as the strongly negatively correlated genes (*n* = 14) from the AKTi+ARi *vs*. t-NEPC GSEA analysis were stratified and analyzed by IPA for GO categorizations. Differential expressions of these genes were presented in the heatmap. **C.** Within the t-NEPC gene set, REST-regulated genes (fold change > 1.5 and padj < 0.1) were stratified and their differential expressions were shown in the heatmap (left). The number of REST-regulated and non-REST regulated genes from Group A_LEAD and Group B_LEAD of the GSEA enrichment plots were presented in 2 separate pie charts (right). Difference of the proportions of REST-regulated genes within the leading-edge groups was calculated by chi-square test.

We further compared the transcriptomes of AKTi, ARi, or AKTi+ARi with the top 200 upregulated genes (ranked by fold change and filtered with padj < 0.05) specific to t-NEPC patients from the Beltran 2016 cohort [12]. GSEA analyses showed that the transcriptome of AKTi alone did not significantly correlate with these upregulated genes specific to t-NEPC patients (FDR = 0.715), while the transcriptomes of ARi (FDR = 0.004) and AKTi+ARi (FDR = 0.002) did (Figure 5A). Interestingly, GO term categorization of the positively and negatively correlated genes within the t-NEPC gene set by Ingenuity Pathway analysis (IPA) revealed that the positively correlated genes were mostly related to neurogenesis, while the negatively correlated genes were mainly related to cell proliferation across the AKTi, ARi, AKTi+ARi groups (Supplementary Figure S7). For example, the leading edge subset genes (*n* = 43, defined as the core subset of genes responsible for the enrichment score calculation [33]) from the AKTi+ARi GSEA analysis (Group B_LEAD) were mainly associated with synapse, neurodevelopment, and molecular transport (Figure 5B). In contrast, the strongly negatively correlated core subset of genes (*n* = 14) (Group B_DOWN) were mainly related to cell growth and proliferation. Consistent with what we have observed in the LNCaP models, combination inhibitions of AKTi and ARi not only increase the diversity but also the fold changes of t-NEPC specific genes. For example, genes such as EYV5, SYP, SYT4, and DCC were mainly upregulated by either AKTi or ARi alone. Genes such as DLGAP3, CCDC151, and HCN3 were more strongly upregulated by the combination AKTi+ARi treatment compared to each of the single treatments (Figure 5B). These findings indicate that combination of AKTi and ARi contributes to NE phenotypes of t-NEPC, but not the highly proliferative properties of t-NEPC.

The significance of REST for t-NEPC progression is evidenced by that there are 29 REST-regulated genes within the top ranked 200 upregulated genes specific to t-NEPC patients. All 29 genes were upregulated by AKTi plus ARi, while only 20 genes were upregulated by AKTi and 26 were upregulated by ARi alone (Figure 5C, left). Genes such as DISP2, AP3B3 and MANEAL were more strongly upregulated by combination treatment of AKTi and ARi. In addition, within the leading edge subgroup of the ARi *vs* t-NEPC GSEA analysis (Group A_LEAD, Figure 5C, right), 22% (12/53) genes were regulated by REST. In comparison, 49% (21/43) genes in the leading edge subgroup of the ARi+AKTi *vs* t-NEPC GSEA analysis (Group B_LEAD) were regulated by REST (*p* = 0.0135) (Figure 5C, right). Together, these findings from clinical t-NEPC patient samples suggest that AKTi can further enhance ARi induced t-NEPC development *via* abolishing the suppressive functions of REST.

## DISCUSSION

**Figure 6:**
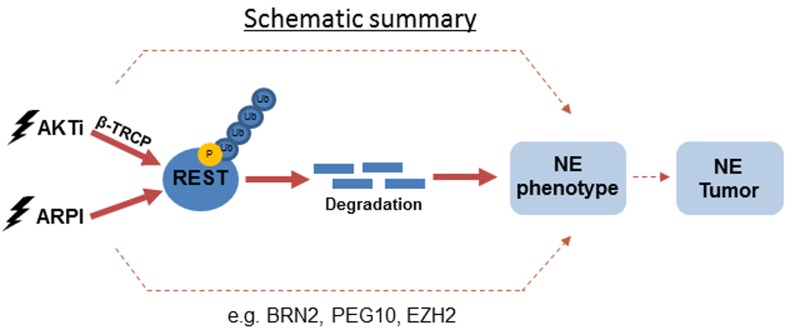
A schematic diagram showing the proposed mechanisms of REST degradation upon PI3K/AKT inhibition and ARPI

Although NEPC is rare in untreated PCa patients, the stringent hormone therapies now used for advanced PCa/CRPC are associated with a significantly increased risk for the development of t-NEPC [34]. In fact, some estimate that up to 25% of patients treated with enzalutamide or abiraterone will develop t-NEPC [11]. NEPC is highly aggressive and is particularly difficult to treat. Current strategies for treatment of NEPC are based on the use of a platinum-based agent in conjunction with etoposide [10]. This therapy, unfortunately, only provides palliative relief. Given the increasing rate of occurrence of t-NEPC in these cohorts, it would be prudent to assess whether novel therapeutic agents used to treat CRPC might make the situation worse. In this study, we assessed the possibility that novel PI3K/AKT-targeted therapies for PCa might also contribute to the development of t-NEPC. Here, we identified REST as a novel downstream effector of PI3K/AKT signaling (Figure 6) and showed that inhibitors of PI3K/AKT reduce expression of REST protein in androgen-sensitive and -insensitive PCa cells. PI3K/AKT inhibition enhanced REST protein degradation through a β-TRCP mediated proteasome pathway, which in turn induced an NE-like phenotype in the treated PCa cells. We showed that the combination of AKTi and ARi can further aggravate REST depletion and promote NE transdifferentiation of PCa cells.

Loss of REST is a key factor for prostate adenocarcinoma cells to gain NE phenotypes under various conditions [35-37], including AR inhibition [22, 23]. Because of the reciprocal activation mechanism between PI3K/AKT and AR signaling pathways [7], we originally hypothesized that PI3K/AKT inhibition would increase AR function in PTEN-deficient cells, thereby stabilizing REST expression to prevent neuroendocrine differentiation. On the contrary, we found that PI3K/AKT inhibition by either AKT siRNA or PI3K inhibitors (LY294002 and BKM120) also downregulated REST protein levels. Our findings also indicate that REST depletion by PI3K/AKT inhibition is independent of AR activity. REST depletion by PI3K/AKT inhibition relies on the serine phosphorylation of REST, indicating that these serine residuals are not directly targeted by AKT. It is possible that other kinases such as CK1 are activated by AKTi to subsequently phosphorylate REST [38]. We have also shown that PI3K/AKT inhibition can induce β-TRCP expression, which in turn recognizes phosphorylated forms of REST for ubiquitination and proteasome degradation. Together, these findings led us to conclude that REST is a novel downstream effector of the PI3K/AKT signaling and that blocking the PI3K/AKT signaling confers PCa cells an NE phenotype *via* REST protein degradation.

Both PI3K/AKT inhibition and ARi alone downregulated REST expression, but the combination treatment resulted in additive suppression of REST protein levels and induction of REST-regulated NE genes. Although AKTi and ARi all exert their actions through β-TRCP, our results (Supplementary Figure S4) as well as others [20] showed that REST reduction by ARi requires at least 96 hours, while PI3K/AKT inhibition induced a more rapid reduction of REST in 8-48 hours. These findings suggest that PI3K/AKT inhibition and ARi may utilize different mechanisms to trigger β-TRCP to mediate REST ubiquitination. One possibility is that PI3K/AKT inhibition may enhance serine phosphorylation of REST that promotes REST ubiquitination. Combined with upregulation of β-TRCP that we observed, then, PI3K/AKT inhibition could induce more rapid REST degradation than ARi does. In addition, PI3K/AKT inhibition and ARi may exert different but complex impacts on REST functions because AR is a nuclear transcriptional factor that can form a protein complex with REST and regulate REST transcriptional activities [20] whereas AKT is a cytoplasmic kinase that mediates signal cascades and may indirectly affect REST functions. This was evident in that although REST siRNA knockdown upregulates both SYP and NSE (Supplementary Figure S1), AKTi only upregulates SYP while ARi induces NSE despite both treatments reduce REST expression (Supplementary Figure S8). These different impacts on REST functions by AKTi and ARi, as a result, may explain why AKTi+ARi upregulates a broader spectrum of REST-regulated genes compared to single treatments both in the LNCaP cell model (Figure 4C) and in the context of t-NEPC patient tumors (Figure 5C). Regardless of the differences, these findings all support that the combination treatment of AKTi and ARi will result in a stronger NE phenotype of PCa cells.

Although the transcriptomes of AKTi and ARi were highly associated with REST siRNA knockdown in the LNCaP cell model, the AKTi transcriptome was not significantly correlated with the genes specific to t-NEPC patients (Figure 5A). Patients in the t-NEPC cohort had not received PI3K/AKT treatment and 33.3% of these patients also had PTEN deletions that resulted in overactive AKT [12], let alone the reciprocal activation of the PI3K/AKT pathway under anti-AR therapies. In contrast, the positive correlation of the ARi transcriptome with the t-NEPC gene set (Figure 5A) is consistent with that these t-NEPC tumors had undergone anti-AR therapies and are likely therapy-induced. However, AKTi plus ARi induced a broader and stronger t-NEPC specific gene changes (Figure 5B) and REST-regulated gene expressions (Figure 5C), suggesting that treatment of PI3K/AKT inhibition to a PCa patient may facilitate the progress of ARi-induced t-NEPC tumor development. Furthermore, since AKTi and ARi mainly regulate different transcriptomes (Figure 4D), we expect that the combination of AKTi with ARi could potentially induce uncharacterized subtypes of t-NEPC under the selection pressure that may be different from the 6 proposed subtypes of t-NEPC tumors [39]. While the traditional role of PI3K/AKT signaling pathway was to promote cell survival and proliferation, overexpression of AKT has been implicated in t-NEPC development. For example, recent reports have shown that N-Myc/AKT overexpression and Rb1/PTEN knockdown can induce neuroendocrine tumors in transgenic mice and xenografts [14, 16, 17]. Interestingly, the NE tumors developed from the Lee et al. study were derived from basal epithelial cells, where AR-negative basal and neuroendocrine cells reside [14]. The NE-phenotype in tumors developed from the Darleen et al. group required N-Myc overexpression while AKT overexpression alone was not sufficient [17]. Similarly, PTEN knockdown initiated only metastatic adenocarcinomas but not NEPC as reported by Ku et al [16]. Based on these findings, we propose that depending on the initial phenotype (luminal epithelial or neuroendocrine) of PCa cells, gain-of-function of AKT can stimulate cell proliferation that drives either AdPC or NEPC tumor formation. On the other hand, AKT blockade in PTEN-deficient PCa cells not only suppresses proliferation, but also induces neuroendocrine transdifferentiation through down-regulating REST expressions.

Recent findings support that t-NEPC is likely derived from adenocarcinoma (AdPC) through coordinated neuroendocrine differentiation and cell proliferation processes under the selection pressure of ARPI [19]. While AR blockade is necessary for t-NEPC establishment by inducing neuroendocrine differentiation, this process is insufficient since only about 25-30% ARPI treated tumors are transformed into t-NEPC [11]. Cancer cells acquired neuroendocrine phenotypes have to further possess or gain a proliferative state to allow t-NEPC tumor establishment. Consistent with our proposed hypothesis, we observe in this study that PI3K/AKT inhibition may provide an opportunity for PTEN-deficient PCa cells to gain an NE-phenotype by downregulating REST (Figure 5B) while inhibiting cell growth and proliferation. These findings imply that PI3K/AKT inhibition can play an important role in initiating neuroendocrine differentiation, a putative early event necessary for t-NEPC tumor establishment.

In summary, we report a novel finding that blocking the PI3K/AKT signaling pathway can reduce REST and induce NE phenotypes in PTEN-deficient PCa cells. Co-targeting PI3K/AKT and AR resulted in more REST depletion and stronger neuroendocrine differentiation of PCa cells. These findings indicate a potential implication of PI3K/AKT inhibition in PCa and provide a caution for the development of this therapeutic strategy.

## MATERIALS AND METHODS

### Materials

R1881, DHT, LY294002, BKM-120, MK-2206, rapamycin, MG132, and cyclohexamide were purchased from Cedarlane (Burlington, ON, Canada). Other chemicals, solvents, and solutions were obtained from Sigma-Aldrich (Oakville, ON, Canada).

### Prostate cancer cell lines

LNCaP and PC3 cell lines were purchased from American Type Culture Collection (ATCC; Manassas, VA, USA). LNCaP95 cells were generously gifted from Dr. Alan Meeker of Johns Hopkins University. LNCaP cells were cultured in RPMI-1640 medium with 10% fetal bovine serum (FBS). PC3 cells were cultured in DMEM medium with 10% FBS. LNCaP95 cells were cultured in phenol-free RPMI-1640 medium with 10% charcoal-stripped serum (CSS) (Hyclone).

### Real-time qPCR and immunoblotting

Real-time qPCR assays were performed as previously described [40]. Experiments were carried out with three technical replicates and three independent biological replicates. Immunoblotting assays were performed as we reported [41]. Experiments were repeated in three independent experiments and one representative result was shown. Information on primers and antibodies are listed in Supplementary Table 1. Image J software was used to perform densitometry analyses of protein bands.

### RNA silencing and DNA transient transfections

Cells were transfected with control siRNA (Dharmacon) and siRNA targeting AKT1/2 (cat#.sc-43609, Santa Cruz) using Lipofectamine 3000 according to the manufacturer’s protocol. Transient DNA plasmid transfections also used Lipofectamine 3000. Detailed information on plasmid DNA, siRNA, and chemicals is listed in Supplementary Table.

### *In vivo* ubiquitination assay

*In vivo* ubiquitination assays were performed as previously described [42] with a modification that includes the application of 1% SDS to induce a denatured condition. Cells were transfected with plasmids encoding ubiquitin plus REST and its mutants (S1024A, S1027A, S1030A and S1024/1027/1030A) from Drs. Stephen Elledge, Gail Mandel, and Gerald Thiel. Twenty-four hours post-transfection, cells were treated with 8 μM of MG132 with either vehicle or 50 μM of LY294002 for 8 hours. Whole cell lysates were extracted using an NETN buffer (50 mM of Tris pH8.0, 150 mM of NaCl, 1% NP40, 1 mM EDTA) plus phosphatase inhibitors (Roche). Lysates were added with 1% SDS and heated at 95 °C for 5 minutes. Protein extracts were then diluted 10 times before being subjected to immunoprecipitation of either REST or ubiquitin. Precipitated proteins were then immunoblotted to detect protein of interest.

### Luciferase assay

LNCaP cells were transfected with plasmids including: wildtype REST or REST with tri-serine degron mutations, SYN-luciferase reporter with wildtype RE-1 or SYN-luciferase reporter with RE-1 loss-of-function mutation, and the renilla reporter as a control for transfection efficiency. Luciferase activities were measured by using the luciferin reagent (Promega, Madison, WI) according to the manufacturer’s protocol. Transfection efficiency was normalized by renilla luciferase activity.

### Immunofluorescence assay

LNCaP were fixed in 4% paraformaldehyde and permeabilized in 0.25% Triton X-100, followed by blocking in 3% milk and overnight incubation with the SYP antibody at 4 °C. The cells were then washed with PBST (PBS with 0.1% Triton X-100), and incubated with the FITC-conjugated secondary antibody (1:1000 in PBST containing 1% milk). After washing with PBST, the cells were mounted in DAPI mounting media (Sigma-Aldrich (Oakville, ON, Canada). Cell imaging was captured by an Axio Observer Z1 Microscope (Carl Zeiss, Thornwood, NY).

### Gene profiling

LNCaP cells that were treated with control, AKTi, ARi and AKTi+ARi and RNA was extracted by using the mirVana RNA Isolation Kit (Ambion, Burlington, Canada) according to the manufacturer’s protocol. Two independently repeated experiments were performed for each experimental condition. The quantity and quality of the RNA samples were assessed by Nanodrop 2000 as well as Agilent 2100 Bioanalyzer (Caliper Technologies Corp., Canada) before sent for AmpliSeq Transcriptome Sequencing. Library preparation, sequencing, and primary analyses were performed by the UBC-DMCBH Next Generation Sequencing Centre following the protocol described by Li *et al* [32]. In summary, cDNA was synthesized from 100 ng of total RNA using the SuperScript^®^ VILO™ cDNA Synthesis kit and amplified with Ion AmpliSeq™ technology. Barcoded cDNA libraries were diluted to 100pM, equally pooled, and amplified on Ion Torren OneTouch2 instrument using emulsion PCR. Then, templated libraries were subjected for sequencing of > 20,000 RefSeq transcripts using the Ion Torrent Proton™ sequencing system. Primary analysis and normalization were performed using the AmpliSeq RNA plugin available through the Ion Torrent™ suite Software [32].

### Bioinformatics and statistical analyses

Differential Gene Analysis (DEG) was performed using R/Bioconductor package DESeq2 with Ampliseq raw counts [43]. Statistical analyses were carried out using R (version 3.3.2) for parametric (2-tailed student’s paired or unpaired *t*-test, and one-way ANOVA test followed by Tukey’ post-hoc test) with statistical significance set at *p* < 0.05 as *, *p* < 0.01 as ** and *p* < 0.001 as ***, and non-parametric (chi-square test) statistics.

## SUPPLEMENTARY MATERIALS FIGURES AND TABLES




