# Correction: Implications of PI3K/AKT inhibition on REST protein stability and neuroendocrine phenotype acquisition in prostate cancer cells

**DOI:** 10.18632/oncotarget.25759

**Published:** 2018-07-03

**Authors:** Ruiqi Chen, Yinan Li, Ralph Buttyan, Xuesen Dong

**Affiliations:** ^1^ Vancouver Prostate Center, Department of Urologic Sciences, The University of British Columbia, Vancouver, Canada

**This article has been corrected:** The corrected author name is given below:

**Ruiqi Chen**

Original article: Oncotarget. 2017; 8:84863-84876. https://doi.org/10.18632/oncotarget.19386

