# Supplementary material for: Implications of PI3K/AKT inhibition on REST protein stability and neuroendocrine phenotype acquisition in prostate cancer cells
Source: Oncotarget. 2017 Jul 19;8(49):84863–76. doi: 10.18632/oncotarget.19386 (PMC5689579; doi:10.18632/oncotarget.19386)
Supplement: Supplementary file 1 [file oncotarget-08-84863-s001.pdf]

Implications of PI3K/AKT inhibition on REST protein stability and neuroendocrine phenotype acquisition in prostate cancer cells

SUPPLEMENTARY MATERIALS

For Supplementary Tables see in Supplementary Files

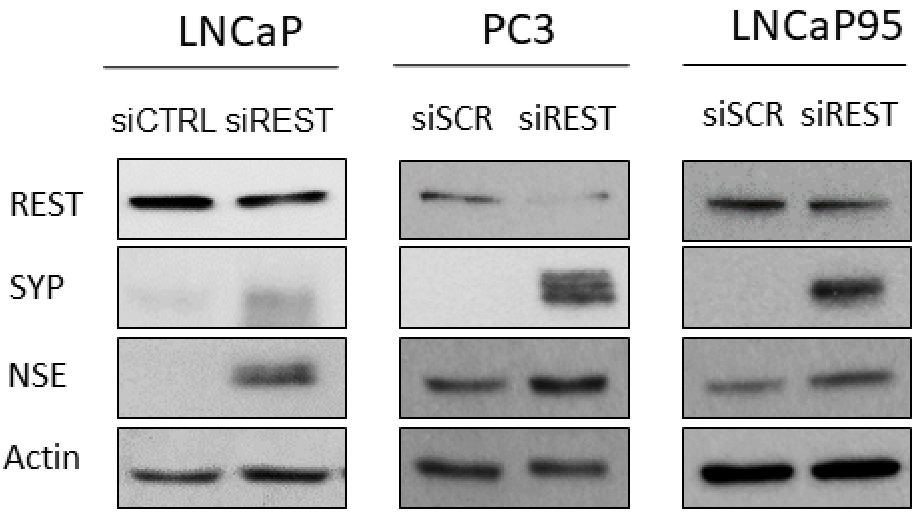

Supplementary Figure S1: LNCaP, PC3, and LNCaP95 cells were transfected with siRNA against control or siRNA against REST. Cell lysates were collected and REST, NSE, SYP, and β-actin protein levels were measured by immunoblotting. Experiments were repeated at least three times and one set of the representative blots was shown.

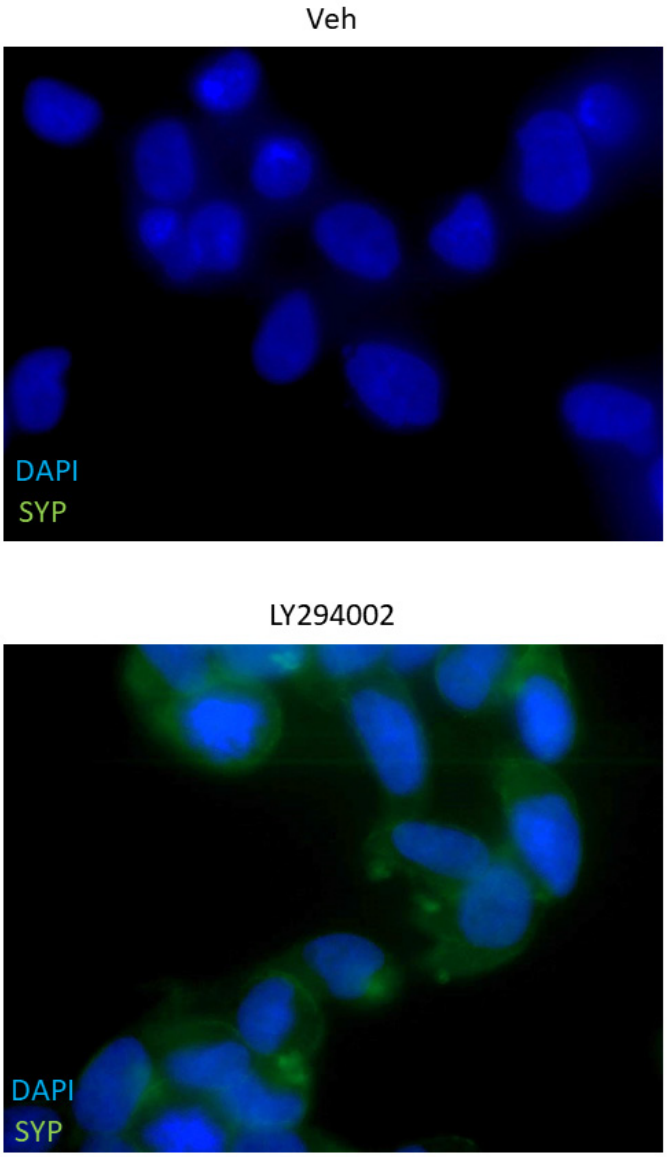

Supplementary Figure S2: LNCaP cells were treated with either Veh or 50 µM LY294002 for 24 hours. Cells were fixed, immunostained with the SYP (D4) antibody, and examined by fluorescence microscope.

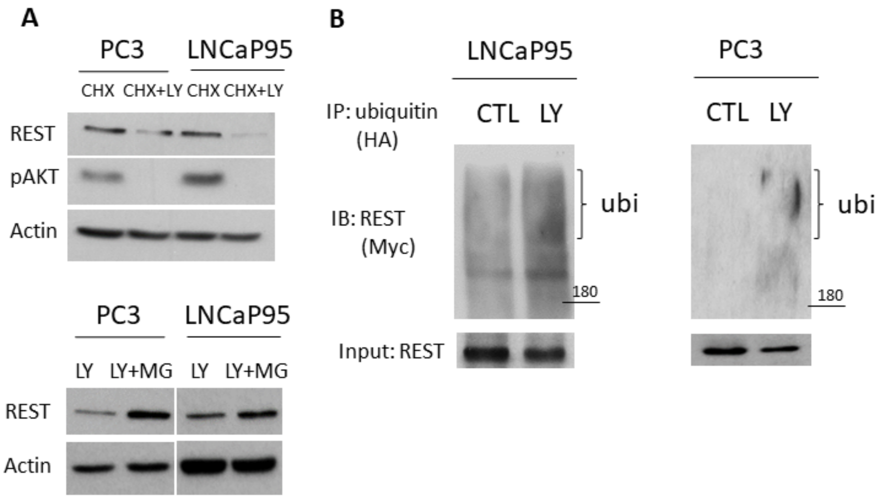

Supplementary Figure S3: (A) PC3 and LNCaP95 cells were treated with 100 ug/ml cyclohexamide (CHX) in the condition of vehicle or 50 µM LY294002 for 24 hours (Top). PC3 and LNCaP95 cells were also treated with 50 µM LY294002 in the condition of vehicle or 8 µM MG132 for 16 hours (bottom). Cell lysates were immunoblotted with antibodies against REST and β-actin. Experiments were repeated at least three times and one set of the representative blots was shown. (B) LNCaP95 and PC3 cells were transfected with flag-tagged wildtype REST and HA-tagged ubiquitin for 48 hours followed by 8 µM MG132 plus vehicle or 50 µM LY294002 treatment for 8 hours. Cell lysates were immunoprecipitated with the HA antibody followed by immunoblotting of the myc antibody for ubiquitinated-REST detection.

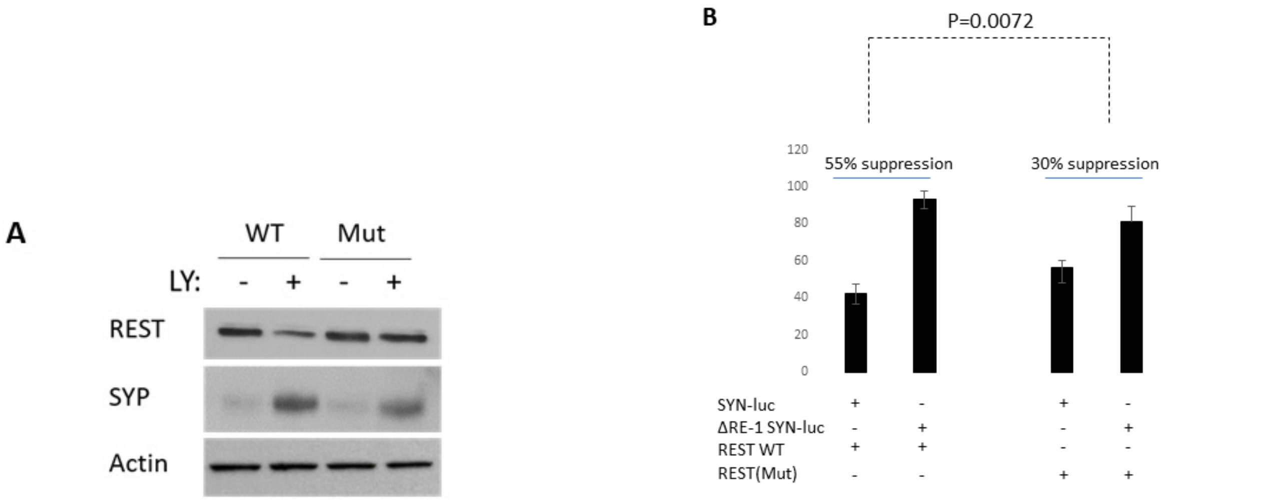

Supplementary Figure S4: (A) LNCaP cells were transfected with wildtype REST (WT) or REST with tri-serine degron mutation (Mut) for 24 hours followed by veh or 50 µM LY294002 treatment for 24 hours. Cell lysates were collected and immunoblotted with REST, SYP, and β-actin. Experiments were repeated at least three times and one set of the representative blots was shown. (B) LNCaP cells were transfected with wildtype REST or REST with tri-serine degron mutations, SYN-luciferase reporter with wildtype RE-1 or SYN-luciferase reporter with RE-1 loss-of-function mutation, and the renilla reporter. REST function was measured by luciferase activities. Statistical analyses were performed by 2-tailed student's paired t-test.

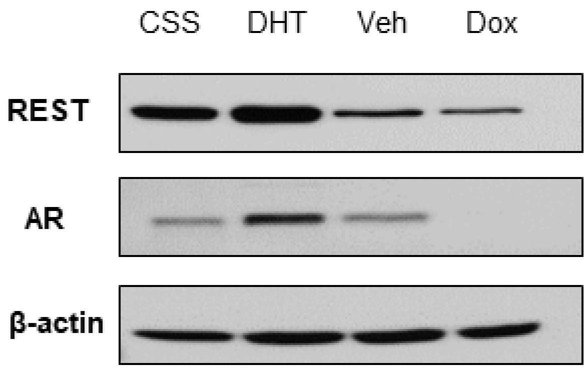

Supplementary Figure S5: Parental LNCaP cells were cultured under CSS for 2 days followed by the addition of vehicle or 10 nM dihydrotestosterone (DHT) for 2 days. Doxycycline-inducible LNCaP(shAR) cells44 were cultured in normal FBS medium followed by the treatment of either vehicle (Veh) or 2 nM doxcycycline (Dox) for 48 hours. Cell lysates were collected and REST, AR, and β-actin protein levels were measured by immunoblotting.

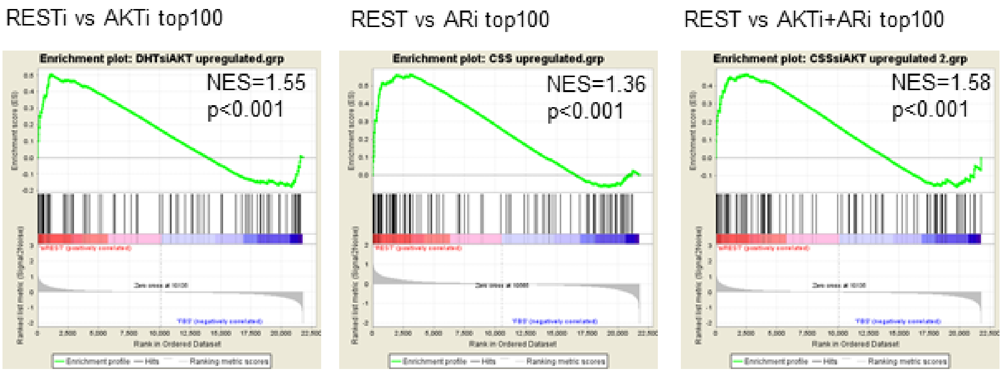

Supplementary Figure S6: GSEA analyses showing correlations of RESTi (GSE51463) with the top 100 upregulated genes from the AKTi, ARi, AKTi+ARi groups. Top 100 upregulated genes were ranked by log2 fold change and filtered with padj<0.1.

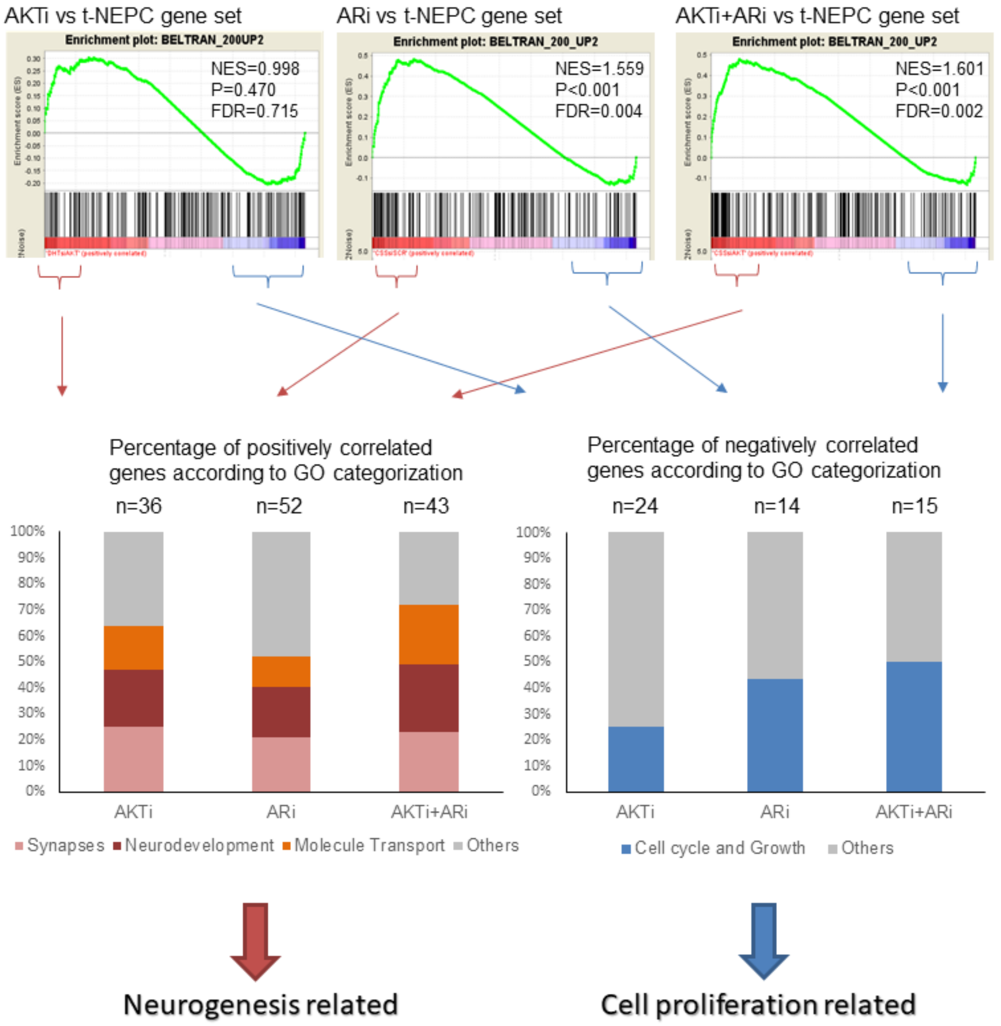

Supplementary Figure S7: The positively correlated leading-edge group and negatively correlated genes from the GSEA analyses including AKTi vs t-NEPC, ARi vs t-NEPC, and AKTi+ARi vs t-NEPC shown in Fig. 5A were stratified and categorized by DAVID GO_TERM analyses (version 6.7, http://david.abcc.ncifcrf.gov/). For positively correlated genes, groups of synapse, neurodevelopment, molecule transport, and others were presented as the percentage of the total numbers (n) of genes being sorted. Similarly, for negatively correlated genes, cell cycle and proliferation-related genes were represented as a percentage of the total numbers (n) of genes being sorted. Whole list and categorization of these genes were available in Supplementary Table S2.

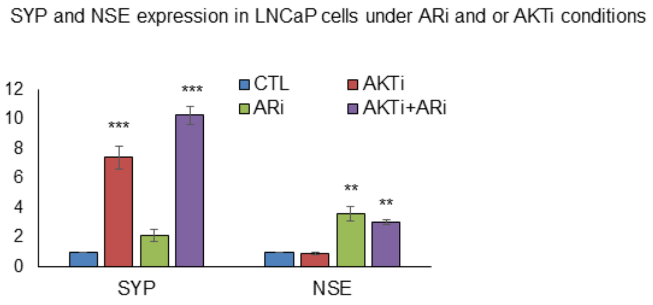

Supplementary Figure S8: LNCaP cells were treated with either control (CTL), AKTi, ARi, or AKTi+ARi. Real-time PCR results of the relative mRNA expressions of SYP and NSE were shown. Statistical analyses were performed by one-way ANOVA followed by Tuckey's post-hoc test with $p < 0.01$ as ** and $p < 0.001$ as ***.
