# Supplementary material for: Implications of PI3K/AKT inhibition on REST protein stability and neuroendocrine phenotype acquisition in prostate cancer cells
Source: Oncotarget. 2017 Jul 19;8(49):84863–76. doi: 10.18632/oncotarget.19386 (PMC5689579; doi:10.18632/oncotarget.19386)
Supplement: Supplementary file 2 [file oncotarget-08-84863-s002.docx]

**AKT inhibition induces neuroendocrine phenotype in prostate cancer cells via REST protein degradation**

**I. SUPPL. TABLE 1: Primers for real-time qPCR**

| **Gene Name** | **Sequence** |
| --- | --- |
| GAPDH F | 5'- GGA CCT GAC CTG CCG TCT AGA A -3' |
| GAPDH R | 5'- GGT GTC GCT GTT GAA GTC AGA G -3' |
| AKT1 F | 5'- TGG ACT ACC TGC ACT CGG AGA A -3' |
| AKT1 R | 5'- GTG CCG CAA AAG GTC TTC ATG G -3' |
| REST F | 5'- GCC GCA CCT CAG CTT ATT ATG-3' |
| REST R | 5'- CCG GCA TCA GTT CTG CCA T-3' |
| CHGA F | 5'- TAA AGG GGA TAC CGA GGT GAT G -3' |
| CHGA R | 5'- TCG GAG TGT CTC AAA ACA TTC C -3' |
| CHGB F | 5'- CGA GGG GAA GAT AGC AGT GAA -3' |
| CHGB R | 5'- CAG CAT GTG TTT CCG ATC TGG -3' |
| ENO2 F | 5’- CCG GGA ACT CAG ACC TCA TC -3’ |
| ENO2 R | 5’- CTC TGC ACC TAG TCG CAT GG -3’ |
| SYP F | 5'- TTA GTT GGG GAC TAC TCC TCG -3' |
| SYP R | 5'- GGC CCT TTG TTA TTC TCT CGG TA -3' |
| NKX3.1 F | 5'- CCC ACA CTC AGG TGA TCG AG -3' |
| NKX3.1 R | 5'- GAG CTG CTT TCG CTT AGT CTT -3' |
| KCNH6 F  KCNH6 R | 5'- GTC GCT CCC CAA AAC ACT TAC-3'  5'- CGA AGA GTT CGC AGA AGC C-3' |
|  |  |
| SYT4 F | 5'- TCCCGAGAGAGGAATTAGAACTT -3' |
| SYT4 R | 5'- TCCCGAGAGAGGAATTAGAACTT -3' |
|  |  |

**II. SUPPL. TABLE 2: Antibodies, siRNA, and A/G beads**

| **Antibody** | **Description** | **Cat No.** | **Supplier** |
| --- | --- | --- | --- |
| Flag | M5 | F4042 | Sigma Aldrich |
| HA |  | ab137838 | Abcam |
| Myc-tag |  | ab9106 | Abcam |
| Beta-actin | C-11 | sc-1615 | Santa Cruz Biotech |
| AR | N-20 | SC-816 | Santa Cruz Biotech |
| REST | EPR2346Y | Ab75785 | Abcam |
| REST |  | 07-579 | Millipore |
| pAKT | Ser473 | 9271 | Cell Signaling |
| AKT |  | 9272 | Cell Signaling |
| SYP | D-4 | Sc-17750 | Santa Cruz Biotech |
| NSE |  | MAB324 | Millipore |
| Beta-TRCP | C-6 | Sc-390629 | Santa Cruz Biotech |
| HAUSP | H-200 | sc-30164 | Santa Cruz Biotech |
| Sg III | C-19 | sc-1492 | Santa Cruz Biotech |
| Control siRNA |  | D-001210-01-20 | Dharmacon |
| AKT1/2 siRNA |  | sc-43609 | Santa Cruz Biotech |
| A/G Agarose beads |  | sc-2003 | Santa Cruz Biotech |
